# Comparative genomics of ParaHox clusters of teleost fishes: gene cluster breakup and the retention of gene sets following whole genome duplications

**DOI:** 10.1186/1471-2164-8-312

**Published:** 2007-09-06

**Authors:** Nicol Siegel, Simone Hoegg, Walter Salzburger, Ingo Braasch, Axel Meyer

**Affiliations:** 1 Lehrstuhl für Zoologie und Evolutionsbiologie, Department of Biology, University of Konstanz, Konstanz, Germany; 2 Department of Ecology and Evolution (DEE), University of Lausanne UNIL Sorge, Le Biophore, Lausanne, Switzerland; 3 Physiological Chemistry I, Biozentrum, University of Würzburg, Germany

## Abstract

**Background:**

The evolutionary lineage leading to the teleost fish underwent a whole genome duplication termed FSGD or 3R in addition to two prior genome duplications that took place earlier during vertebrate evolution (termed 1R and 2R). Resulting from the FSGD, additional copies of genes are present in fish, compared to tetrapods whose lineage did not experience the 3R genome duplication. Interestingly, we find that ParaHox genes do not differ in number in extant teleost fishes despite their additional genome duplication from the genomic situation in mammals, but they are distributed over twice as many paralogous regions in fish genomes.

**Results:**

We determined the DNA sequence of the entire ParaHox C1 paralogon in the East African cichlid fish *Astatotilapia burtoni*, and compared it to orthologous regions in other vertebrate genomes as well as to the paralogous vertebrate ParaHox D paralogons. Evolutionary relationships among genes from these four chromosomal regions were studied with several phylogenetic algorithms. We provide evidence that the genes of the ParaHox C paralogous cluster are duplicated in teleosts, just as it had been shown previously for the D paralogon genes. Overall, however, synteny and cluster integrity seems to be less conserved in ParaHox gene clusters than in Hox gene clusters. Comparative analyses of non-coding sequences uncovered conserved, possibly co-regulatory elements, which are likely to contain promoter motives of the genes belonging to the ParaHox paralogons.

**Conclusion:**

There seems to be strong stabilizing selection for gene order as well as gene orientation in the ParaHox C paralogon, since with a few exceptions, only the lengths of the introns and intergenic regions differ between the distantly related species examined. The high degree of evolutionary conservation of this gene cluster's architecture in particular – but possibly clusters of genes more generally – might be linked to the presence of promoter, enhancer or inhibitor motifs that serve to regulate more than just one gene. Therefore, deletions, inversions or relocations of individual genes could destroy the regulation of the clustered genes in this region. The existence of such a regulation network might explain the evolutionary conservation of gene order and orientation over the course of hundreds of millions of years of vertebrate evolution. Another possible explanation for the highly conserved gene order might be the existence of a regulator not located immediately next to its corresponding gene but further away since a relocation or inversion would possibly interrupt this interaction. Different ParaHox clusters were found to have experienced differential gene loss in teleosts. Yet the complete set of these homeobox genes was maintained, albeit distributed over almost twice the number of chromosomes. Selection due to dosage effects and/or stoichiometric disturbance might act more strongly to maintain a modal number of homeobox genes (and possibly transcription factors more generally) per genome, yet permit the accumulation of other (non regulatory) genes associated with these homeobox gene clusters.

## Background

### The cichlid fish

Cichlids belong to the most diverse and species-rich families of fishes. With an estimated number of more than 3,000 species they alone represent more than ten percent of all fish species. The family Cichlidae belongs to the teleosts that, with more than 26,500 species, are the most diverse lineage of all vertebrates [1]. Cichlids have a Gondwanian distribution and are found in India, Madagascar, South and Central America and Africa and developed a stunning variety of coloration patterns, body shapes, behaviors and trophic as well as ecological specializations within a few millions of years see [2-8]. Their unparalleled diversity made the cichlid species flocks a textbook example for parallel adaptive radiations and explosive speciation [7].

The evolutionary success of the cichlids has been attributed to morphological and behavioral patterns, although the relative importance of different mechanisms – as there will be surely more than one – is still debated. One plausible factor that is at least partly responsible for the cichlids' unique diversity is the complexity of their breeding system and social behavior. Cichlids evolved a variety of brood care strategies and mating systems, and it is likely that female choice with respect to male coloration played an important role during cichlid evolution [2,5,9-11]. Another possible reason for their evolutionary success is the particular architecture of the cichlids' jaw apparatus. They possess two sets of jaws, one oral and one pharyngeal jaw derived from the fifth gill arch. These jaws evolved independently from each other and allow for an immense variety of possible feeding types leading to different diets. Therefore, many different niches could be colonized by cichlids [12]. There is a large amount of behavioral and morphological divergence between different cichlid species in the East African lakes. Yet, rather surprising parallelisms have evolved in species flocks of the different lakes [3,5,8] indicating that the genetic "predisposition" for the modification of these traits might have been already present in the genome of the common ancestor of all the East African cichlid species. We assume that a substantial part of the necessary modifications of the cichlids' genome takes place in the regulatory elements of only a few important genes. To test this hypothesis it would be important to identify those genes of relevance in speciation. As part of this overall research effort we focus here on the ParaHox genes, a sister-cluster of the Hox genes that are crucial in development [13,14]. Here, we report on an investigation of the genomics of the ParaHox C and D paralogons of the cichlid *Astatotilapia burtoni *and present the results of a comparison of some of its genomic features with those of other vertebrate ParaHox clusters.

### Genome duplication, Hox- and ParaHox clusters in vertebrates

It has been suggested that gene- or genome duplications might be important evolutionary mechanisms resulting in new copies of genes, which are then free to accumulate mutations and to evolve new or additional functions [15]. Changes in regulatory elements of duplicated gene copies could, for example, cause neofunctionalization; the gain of a new function, or a subfunctionalization; i.e., subdividing the original functions of the duplicated gene between the daughter genes [16]. Genes under relaxed selection can arise after the duplication of single genes, large chromosomal fragments or even whole genomes [[17] and references therein]. For each of these three possibilities, different effects are characteristic: the preservation or disruption of regulatory control, the genomic context, the potential for dosage imbalance and, of course, the size of the duplicated fragment [18].

Duplications of genes as a consequence of the activity of transposable elements, unequal crossing-over and other mechanisms occur frequently in the course of vertebrate evolution [19]. The duplication of whole genomes, however is a rare event in animals, although there are quite a few polyploid species in some taxonomic groups such as frogs [20], salamanders [21] and several fish lineages such as salmonids [22], cyprinids and catfish [23]). In plants polyploidy is a rather common phenomenon [24-26].

Several studies have proposed the existence of two rounds of whole genome duplications during vertebrate evolution (2R hypothesis) [[14,27] and references therein]. More recent analyses revealed that in the lineage leading to the ray-finned fish, an additional genome duplication event, the fish-specific genome duplication (3R or FSGD), has occurred [28-33]. The 1R and 2R can be roughly dated 430 – 750 mya [27,34] in the lineage of the Gnathostomata. However, the phylogenetic relationships of the agnathan lineages to one other and to the vertebrates as well the timing of 1R and 2R is not fully resolved yet [35]. The FSGD [36,37] took place in the lineage of ray-finned fish, after the separation of gars but before the origin of the Osteoglossomorpha [30], around 320 mya [27,38].

Among the first to be discovered and still among the most prominent examples for duplicated genes through whole genome duplications are the Hox clusters [14]. The number of Hox gene clusters and their genomic architecture in vertebrate genomes are an excellent illustration for the vertebrate genomic history of two rounds of genome duplications (1R, 2R), as well as an additional fish specific genome duplication (3R/FSGD) [39]. One cluster is found in the genome of the Cephalochordate *Branchiostoma *and one cluster is assumed to be the ancestral state [40]. Two rounds of genome duplication led to four copies in sharks and tetrapods and another round of genome duplication along with reciprocal losses of genes lead to a total number seven Hox clusters in teleost fish [33,39]. Therefore it might be expected that the genes of the ParaHox clusters, just as those of the Hox clusters, should reflect the history of the last two genome duplications in fish as well [41].

**Figure 1:**
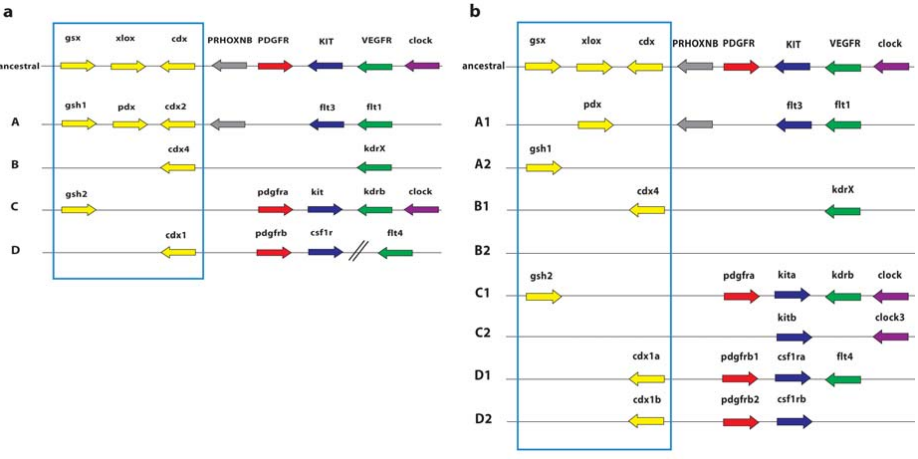
**Schematic of the genomic architecture of the vertebrate ParaHox loci**. **a: **Structure of the presumed ancestral vertebrate condition and the mammalian ParaHox clusters and their 3' adjoining genes ("ParaHox paralogon"). **b: **structure of the derived (post FSGD) teleost fish ParaHox cluster genomic architecture and their 3' adjacent genes [43-45] The color code of the relevant genes is maintianed throughout this paper: yellow: ParaHox genes, red: *pdgfr *genes, blue: *kit *and *csf1r *genes, green: *vegfr *genes, purple: *clock *genes; the colors red, blue and green also stand for RTKs type III.

The ParaHox complex in mammals consists, just as the Hox complex, out of four clusters termed A to D. But only the A cluster still carries all three genes of the predicted ancestral ParaHox cluster. All other clusters contain only a single ParaHox gene at most. The ParaHox complex is even more fragmented in teleost fish, and Mulley et al. [42] argued that there are no teleost ParaHox clusters at all. However, if the adjoining genes of the ParaHox clusters are taken into account, the syntenic structure of the genomic region/paralogon that contains the ParaHox gene(s) becomes apparent [43-45] (see Figure 1) and the evolutionary history of the clusters can be reconstructed.

The present study regards a ParaHox paralogon as the ParaHox gene(s) from a cluster together with the respective 3' adjoining genes as a [46] (see Figure 1). Therefore, genes located 5'of the whole paralogon are referred to as 5'of a gene X and genes more towards the 3'end of a paralogon are referred to as 3'of a particular gene X, irrespective of the orientation of gene X.

**Figure 2:**
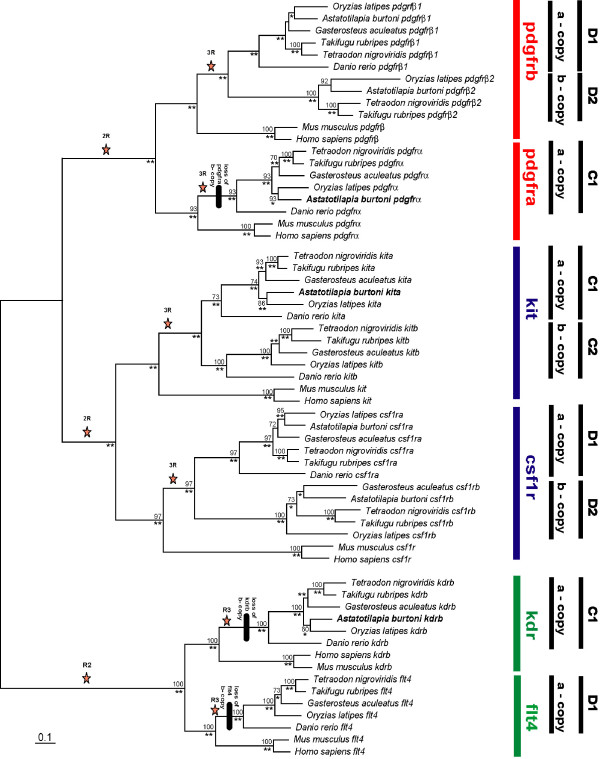
**Phylogenetic analysis of the RTKs of the C and D ParaHox paralogon**. Bayesian analysis, **: 1.00; *: 0.95 – 0.99 support, nucleotide data, stars mark the postulated phylogenetic timing of whole genome duplications. Tree are derived from a PHYML analysis.

For this study we conducted an analysis of the genomic evolution of vertebrate ParaHox paralogons. Specifically, we were interested in the ParaHox clusters C and D and the adjoining type III receptor tyrosine kinase genes (*pdgfrα/kit and pdgfrβ/csf1r *respectively) that were shown to be involved in teleost coloration [46-49]. To this end, we determined the DNA sequence of the entire ParaHox C1 paralogon in the East African cichlid fish *Astatotilapia burtoni*, and compared it to orthologous regions in other teleosts' genomes (*Danio rerio, Oryzias latipes, Tetraodon nigroviridis, Takifugu rubripes, Gasterosteus aculeatus*) and mammals (*Mus musculus, Homo sapiens*) as well as to the *Astatotilapia burtoni *ParaHox D paralogons [46]. This was done in order to investigate the genomic consequences of several rounds of genome duplication in the vertebrate lineage (Figure 2).

## Results and discussion

To investigate the evolution of the vertebrate ParaHox paralogons C and D we shotgun sequenced a BAC clone of a BAC library of the East African cichlid fish *Astatotilapia burtoni *[50] that contained the C1 ParaHox paralogon, i.e., the ParaHox gene *gsh2 *and its 3'adjoining genes. The obtained BAC contig (GenBank accession EF526075 GenBank accession number: sequence will be submitted upon acceptance of the paper) was then further analyzed and compared to the sequences of two other BAC clones of the African cichlid *Astatotilapia burtoni*, 20D21 (DQ386647) and 26M7 (DQ386648) containing the D1 and D2 paralogons [46].

### Sequence assembly and analysis

**Figure 3:**
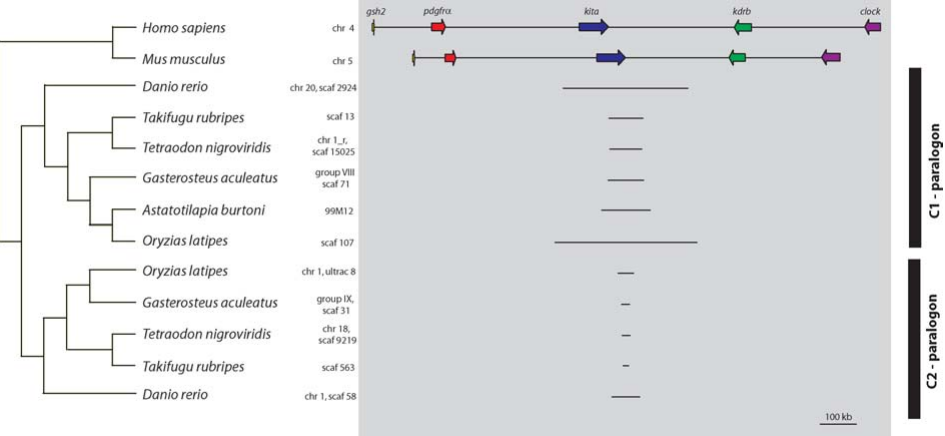
**C1 and C2 ParaHox paralogon – overview**. The C ParaHox paralogon of *Homo sapiens*, *Tetraodon nigroviridis*, *Takifugu rubripes*, *Oryzias latipes*, *Danio rerio, Gasterosteus aculeatus *and *Astatotilapia burtoni*. All teleost fishes except *Astatotilapia *are represented with their a- and b-copy. Of *Astatotilapia *only the a-copy is available at present. The orientation of the five genes and their respective paralogs are printed above the genes. The locations of the genes in their respective genome annotation are written on the right side of the name of the species.

This analysis showed that the C(1) ParaHox gene locus and its 3' adjoining genes of *Danio rerio *is located on chromosome 20, of *Takifugu rubripes *on scaffold 13, of *Tetraodon nigroviridis *on 'chromosome1_random', of *Gasterosteus aculeatus *on group VIII, of *Homo sapiens *on chromosome 4, of *Mus musculus *of chromosome 5 and of *Oryzias latipes *on the scaffolds 1,264 (*gsh2*, *pdgfrα*), 578 (*kita*, *kdrb*) and 2,436 (*clock*) (see Figure 3 and see Additional File 1 [Table S1] for details).

Sequences for the C2 and the D ParaHox gene loci and their 3' adjoining genes were also retrieved from the aforementioned databases and aligned by hand. The locations of the different genes in the respective genome assemblies are summarized in Additional File 1.

### Identification and characterization of *Astatotilapia burtoni *ParaHox paralogon containing BAC clones

The BAC library was screened for the C1 ParaHox paralogon gene *kita *as described previously [50]. A PCR screen for the presence of the ParaHox gene *gsh2 *was subsequently performed to identify BAC clones covering the entire C1 ParaHox paralogon. The *kita *and *gsh2 *positive clone 99M12, which was determined to have an insert length of 154 kb, was chosen for further investigation. The BAC clone was shotgun sequenced and BAC contigs were assembled into a scaffold and a complete sequence as described earlier [50].

**Figure 4:**
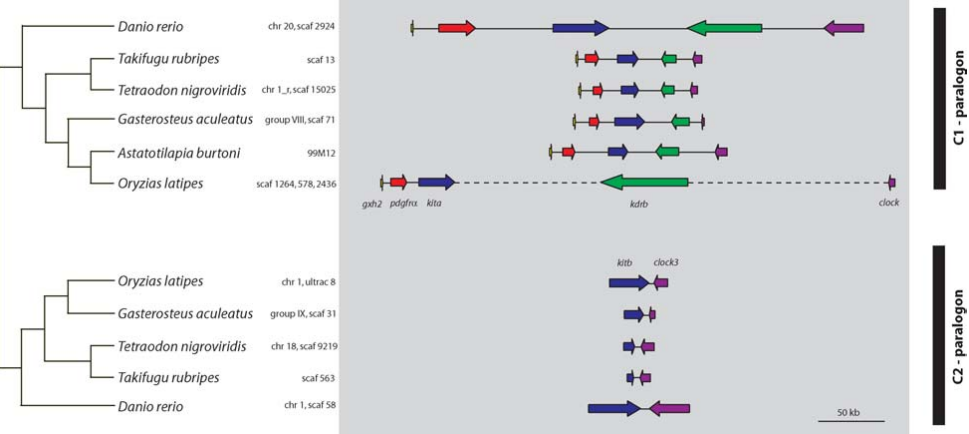
**C1 and C2 ParaHox paralogon**. Detailed depiction of the teleost fish C1 and C2 ParaHox paralogons of Figure 1b. *Homo sapiens *and *Mus musculus *were left out of the figure to ensure a clear image of the genes. The orientation of the genes is indicated by the arrows.

BLAST searches [51] of the assembled contigs against GENBANK [52] showed that five genes were at least partially present in the BAC clone 99M12 (Figures 1 and 4): *genomic screened homeo box 2 *(*gsh2*), the *platelet-derived growth factor receptor α *(*pdgfrα*), the a – copy of the *v-kit Hardy-Zuckerman 4 feline sarcoma viral oncogene homolog *(*kita*), the b – copy of the *kinase insert domain receptor *(*kdrb*) and the *circadian locomoter output cycles kaput *(*clock*) (Figures 1 and 4). The C1 ParaHox paralogon was found to be the paralogon with the highest number of 3' adjoining genes in teleosts.

Using cDNAs, annotated and predicted genes of *Homo sapiens*, *Mus musculus *and *Danio rerio *available on NCBI [52], we deduced the coding sequences of *Takifugu rubripes*, *Tetraodon nigroviridis, Oryzias latipes, Gasterosteus aculeatus *and *Astatotilapia burtoni*. We were able to assemble the complete coding sequences of four of the five genes located on the BAC clone 99M12 of *A. burtoni*. The only incompletely assembled gene is *kdrb *where approximately 200 bp of the coding sequence are missing.

From the beginning of the gene *gsh2 *to the end of *clock *this sequence of the clone 99M12 spans 133.56 kb. This length was used for comparisons of the lengths of the C1 ParaHox paralogons of the different organisms used in this study (*Homo sapiens*, *Danio rerio*, *Takifugu rubripes*, *Tetraodon nigroviridis*, *Oryzias latipes, Gasterosteus aculeatus *and *Astatotilapia burtoni*) because the real length of the inserted gaps is unknown as of present.

**Figure 5:**
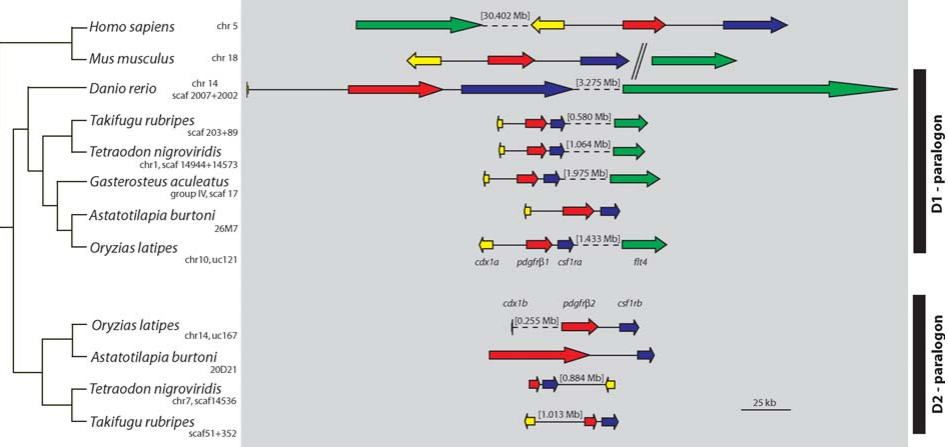
**D1 and D2 ParaHox paralogon**. The D ParaHox paralogon of *Homo sapiens*, *Mus musculus*, *Tetraodon nigroviridis*, *Takifugu rubripes*, *Oryzias latipes, Gasterosteus aculeatus *and *Astatotilapia burtoni*. The orientations of the genes are indicated by the arrows. The chromosomal locations of these paralogons in their respective genomes are indicated next to the species name.

Another BAC clone (26M7) of the *Astatotilapia burtoni *BAC library contains the genes *caudal type homeo box transcription factor 1 a *(*cdx1a*), the *platelet-derived growth factor receptor β1 *(*pdgfrβ1*) and the *colony-stimulating factor 1 receptor a *(*csf1ra*) [46]. These three genes plus the *fms-related tyrosine kinase 4 *(*flt4*) that is not present on the clone belong to the D1 ParaHox paralogon, whereas the clone 20D21 was found to contain the genes *platelet-derived growth factor receptor β2 *(*pdgfrβ2*) and the *colony-stimulating factor 1 receptor b *(*csf1rb*) [46]. These two genes plus the *caudal type homeobox transcription factor 1b *(*cdx1b*) (not contained on this BAC clone) form the D2 ParaHox paralogon. The genes *kitb *and *clock3*, belong to the C2 ParaHox paralogon. Figure 5 shows the D1 and D2 ParaHox paralogons as defined above.

The ancestral ParaHox complex fish is fragmented in teleosts fish [42]. Therefore, we use the expression ParaHox "paralogon" instead of "cluster" since, especially for the case of the C2 ParaHox paralogon, not a single ParaHox gene is still present and in the case of the D2 ParaHox paralogon, the data we investigated did not include the ParaHox *cdx1b *gene. Possibly the most interesting finding is that the ParaHox complex of teleost fish, even after another round of whole genome duplication (WGD), the FSGD, and subsequent deletion of genes, contains exactly the same number of genes and orthologous set of ParaHox genes as the mammalian four ParaHox clusters which did not experience the FSGD. This is all the more surprising since in teleosts all six ParaHox genes are distributed across seven instead of four paralogons, and there is not a single complete ParaHox cluster left in the fish lineage [42]. As outlined above, the ParaHox paralogous genomic regions remain identifiable and we wish to emphasize that the paralogous relationship of the RTKs and other genes 3' of the remnants of those ParaHox clusters stay intact. This is because the remaining genes of the ParaHox clusters, and the 3' adjoining RTKs, as well as the genes *clock *and *clock3 *that lie directly 3' of the RTKs on the C1 and C2 ParaHox clusters respectively, clearly form paralogous genomic regions.

### The C1, C2, D1 and D2 ParaHox paralogons

Using sequence orthology to *Astatotilapia burtoni *we were able to determine the C and D ParaHox paralogons of *Homo sapiens*, *Mus musculus*, *Danio rerio*, *Takifugu rubripes*, *Tetraodon nigroviridis*, *Oryzias latipes *and *Gasterosteus aculeatus *(Additional File 1).

We were unable to find the gene *clock *in the *G. aculeatus genome *except for two very short blast hits. Since each of the genes of the C1 ParaHox paralogon of this species lies on different contigs within one scaffold, it seems likely that this gene was not correctly assembled in the current release of the stickleback genome. The entire D2 ParaHox paralogon of *D. rerio *and a major portion of the expected paralogon of *G. aculeatus *could not be located in the current releases of public genomic databases. The *flt4 *gene of *M. musculus *was relocated to another chromosome and the *flt4 *of *H. sapiens *was relocated to a location 30 Mb 5' of *cdx1*. Therefore it was excluded from the following analyses. The *cdx1b *of *Tetraodon nigroviridis *is relocated as well and the *cdx1b *of *Oryzias latipes *is reversed. All other examined organisms kept the orientations and positions unaltered in reference to the more 5' genes, but the distance to those is always very large (Figure 5).

The orientation and the order of the genes of the C ParaHox paralogons are conserved in all vertebrates species examined (Figures 3 and 4), implying that the orientation and order of these genes have remained unchanged for more than 450 my [53]. The genes *gsh2*, *pdgfrα *and *kita *with its paralogous gene *kitb *all have a 5' – 3' orientation. The genes *kdrb *and *clock *as well as its paralogous gene *clock3*, show a 3' – 5' orientation (Figures 3 and 4, [42,45]). The genomic architecture of the D1 and D2 ParaHox paralogons is less conserved. The orientation of the genes has stayed the same in all but one species included in this study. Yet, in four genomes the position of a gene compared to *pdgfrβa/b *and *csf1ra/b *has changed (Figure 5). Furthermore, the *csf1rb *gene seems to have been lost in *Danio rerio *[46]. The gene content of all other paralogons examined in this study was completely conserved.

The presumed ancestral condition of the C ParaHox paralogon that can still be found in mammalian genomes [43,45], it is also conserved in all teleostean a-copies of this ParaHox paralogon (Figures 1a and 3). In all organisms examined here, the b-copy of the C ParaHox complex has lost genes, namely *gsh2*, *pdgfrα *and *kdrb*. The remaining genes of this paralogon nevertheless retained their orientation. A similar scenario can be seen in the D ParaHox paralogon. Here only the b-copy of the gene *flt4*, a 2R-paralog of *kdrb*, was lost. We found no trace of *clock*-like genes 3' of the RTKs so we can not say whether both *clock *copies were deleted or if the *clock *precursor was located in the C ParaHox paralogon after the precursor of the C and the D ParaHox paralogons was duplicated. This implies that there never was a *clock*-like gene in the D1 and D2 ParaHox paralogons.

It seems quite remarkable, that this gene complex maintained both its gene order as well as gene orientation (with the exception of two genes) over very significant evolutionary time spans. Only the lengths of the introns and intergenic regions differ between the species examined. Possible reasons for this conservation might be related to the presence of promoter, enhancer or inhibitor motifs in that complex that influence more than just one gene, so that an inversion or a relocation of one gene would possibly destroy the regulation of the proteins constructed from this and other genes nearby. If such a co-regulation exists, it might explain the maintenance of the gene order and gene orientation over the course of vertebrate evolution. Chiou et al. [54] showed an example of the important role of clustering in the regulation of the expression of biosynthetic genes in *A. parasiticus*.

Another possibility might be that a regulator is not located immediately next to its corresponding gene but at a distance, and that other genes exist between regulator and corresponding gene. A relocation or inversion in such a case is expected to lead to disruption of the interactions. It has already been shown that regulatory genes or regions lying in a gene cluster are able to control the expression of genes outside of this cluster [55]. Nevertheless, the selective pressures leading to the maintenance of gene clusters are still poorly understood.

In both the C and the D paralogons, only the a-copy retained the ParaHox gene. It was either lost (C2 copy) or relocated (D2 copy, Figure 5). So the b-paralogon in both cases lost more genes than the a-paralogon. Therefore, when comparing the C and the D ParaHox paralogons it is apparent that the a-copies of the ParaHox paralogons C and D of the teleosts are more conserved and show a higher degree of synteny with the mammalian ParaHox paralogons than the b-copies. Interestingly, this finding is similar to the pattern previously found in the Hox clusters [56]. This finding implies that one copy of the paralogon pair evolved faster than the other. That this is always usually the b-copy is explained most easily by the fact that the more conserved (a) copy is much more likely to be discovered and named first.

To further investigate this issue, we performed relative rate tests as described previously [46]. These analyses revealed that the genes of the C paralogons always evolved slower than that of the D paralogons, compared to the human gene (data not shown). Also, we found that the a-paralogon genes always evolved more slowly that the b-copy, except *cdx1*. *cdx1a/b*, that are a part of the D1/D2 paralogons respectively, the b-copy evolved more slowly than the a-copy, for unknown reasons – even though the rest of the paralogon follows the normal trend (see Table 1). In this regard, the 3' end and the 5' end of the D1/D2 paralogons seem to experience different evolutionary forces.

**Table 1:** Nonparametric Relative Rate Tests of the C and D ParaHox paralogons

			Nucleotide Sequence (first and second codon position)			Amino Acid Sequence		
			
species	para-logons	gene^a^	sites	unique differences	signifi-cance^b^	sites	unique differences	signifi-cance^b^
*T. nigroviridis*	Ca-Cb	*kita*	1674	134	*	837	43	*
		***kitb***		176			68	
*T. rubripes*		*kita*	1208	74	***	603	30	***
		***kitb***		140			75	
*O. latipes*		*kita*	1524	163	0.166	761	63	0.391
		***kitb***		189			73	
*G. aculeatus*		*kita*	1450	168	0.628	724	64	0.604
		***kitb***		177			70	
*D. rerio*		*kita*	1864	154	*	931	64	*
		***kitb***		192			93	
*T. nigroviridis*		*clock*	1574	114	***	787	55	***
		***clock3***		217			113	
*T. rubripes*		*clock*	1635	107	***	816	51	***
		***clock3***		222			111	
*O. latipes*		***clock***	1562	177	*	780	112	**
		*clock3*		143			67	
*D. rerio*		*clock*	1592	73	***	795	32	***
		***clock3***		203			116	

*T. nigroviridis*	Da-Db	***cdx1a***	428	121	***	213	62	***
		*cdx1b*		39			15	
*T. rubripes*		***cdx1a***	452	116	***	225	62	***
		*cdx1b*		55			22	
*O. latipes*		***cdx1a***	224	30	*	111	11	0.134
		*cdx1b*		17			5	
*T. nigroviridis*		*pdgfrb1*	1966	222	***	982	90	***
		***pdgfrb2***		328			164	
*T. rubripes*		*pdgfrb1*	2032	249	**	1015	98	***
		***pdgfrb2***		323			173	
*O. latipes*		*pdgfrb1*	1999	254	***	998	97	***
		***pdgfrb2***		405			207	
*A. burtoni*		*pdgfrb1*	2025	216	***	1011	72	***
		***pdgfrb2***		301			158	
*T. nigroviridis*		*csf1ra*	1864	228	***	904	54	***
		***csf1rb***		692			323	
*T. rubripes*		*csf1ra*	1842	187	**	920	59	***
		***csf1rb***		258			105	
*O. latipes*		*csf1ra*	1810	208	**	904	67	***
		***csf1rb***		279			119	
*A. burtoni*		*csf1ra*	1856	192	**	927	62	*
		***csf1rb***		247			93	
*G. aculeatus*		*csf1ra*	1782	183	***	890	65	***
		***csf1rb***		306			141	

^a ^Genes that show statistically significant increase in rate of molecular evolution are indicated in bold.

^b ^χ^2 ^tests: *P < 0.1, **P < 0.01, *** P < 0.001.

**Figure 6:**
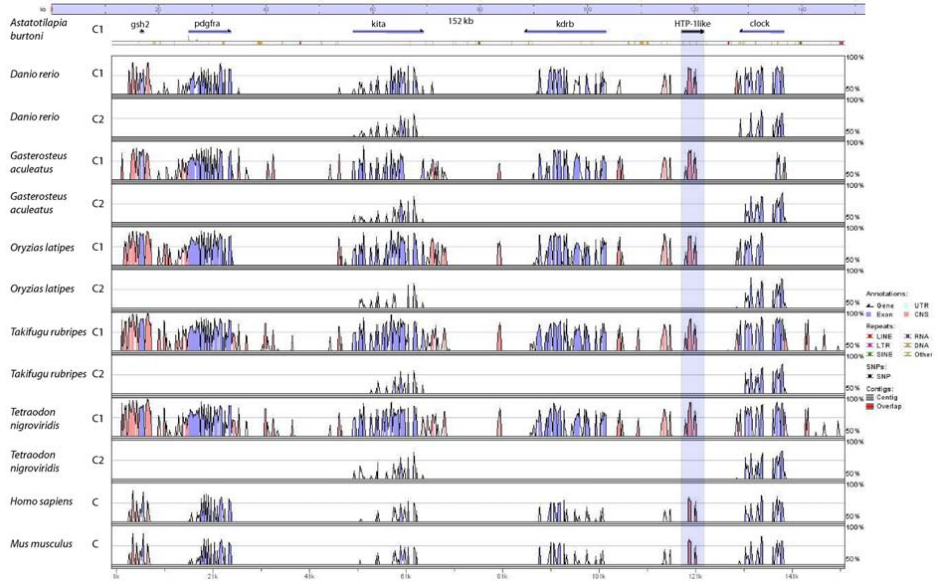
**Shuffle-LAGAN vista blot**. Comparisons of the C1 and C2 ParaHox paralogons from *A. burtoni *(query sequence) with *T. rubripes*, *T. nigroviridis*, *D. rerio*, *O. latipes, Gasterosteus aculeatus, Mus musculus *and *H. sapiens*. The blue peaks are conserved areas in exons of the genes and in pink conserved intergenic regions are indicated. The genes shown on the graph are from left to right: *gsh2*, *pdgfrα*, *kita*, *kdrb *and *clock*.

To search for conserved regions that could possibly be promoter regions, enhancers or inhibitors, we performed a mVista plot analysis comparing the C1 ParaHox paralogon of *Astatotilapia burtoni *with those of *Takifugu rubripes*, *Tetraodon nigroviridis*, *Danio rerio*, *Oryzias latipes, Gasterosteus aculeatus, Mus musculus *and *Homo sapiens*. The genes of these paralogons were, as already mentioned, *gsh*2, *pdgfrα*, *kita*, *kdrb *and *clock *(Figure 6). The results are similar to our previous analyses of the D Parahox paralogons [46]. Conserved intergenic regions immediately upstream of the genes *gsh*2, *pdgfrα *and *kita *can clearly be detected. Furthermore, there are conserved regions also following the gene *clock *as well as between it and *kdrb*. Another conserved region is apparent immediately 3' of *kita *as well as between the genes *gsh2 *and *pdgfrα *(Figure 6). The conserved elements upstream of the genes *gsh2 *and *pdgfrα *and a region between *kdrb *and *clock *are, at least in part, conserved in all vertebrates examined. Through blasting of a subset of the *A. burtoni *sequence against the available databases, we found that the conserved sequences between the genes *kdrb *and *clock*, are similar to another gene, a transmembrane protein called *HTP-1*. The other conserved intergenic regions are only conserved in teleost fish. examined and BLAST searches of those regions of the BAC clone 99M12 revealed them to be SINE and LINE elements. As only the fugu specific mask was available, not all elements could be masked efficiently. The only exception to that pattern is the region in *Oryzias latipes *in the second part of the gene *clock*. Because of problems in the genome assembly of this organism these data could not be used in the mVista blot analysis and, therefore, medaka had to be omitted from this analysis. As already mentioned, the gene *clock *of *Gasterosteus aculeatus *could not be located in its genome assembly. Only two short BLAST hits for *clock *were found with bl2seq (Figure 6).

A comparison of the C1 ParaHox paralogons of *Homo sapiens*, *Astatotilapia burtoni*, *Danio rerio*, *Takifugu rubripes*, *Tetraodon nigroviridis *and *Gasterosteus aculeatus *(Figures 3 and 4) showed that the cichlid sequence is of an intermediate length. It is considerably shorter that *H. sapiens *(10%),*D. rerio *(39%) and *O. latipes *(34%) but longer than *T. nigroviridis *(150%), *T. rubripes *(141%) and *G. aculeatus *(135%) (see Additional file 2). In *O. latipes *only fragments of *clock*, the last gene of the paralogon, could be found. Because of seemingly incomplete assembly in this genomic region 34% might not be the final result.

**Figure 7:**
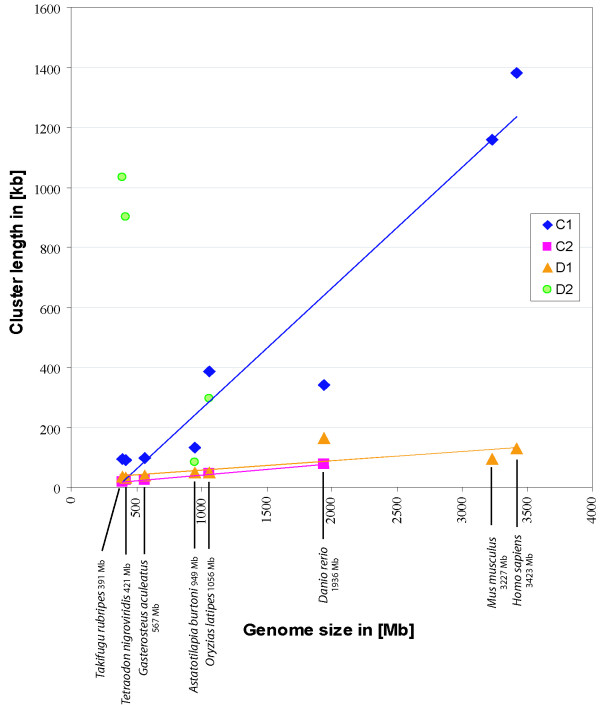
**Comparison (regression analyses) of the genome size and the size of the C1, C2, D1 and D2 ParaHox paralogons**. All genome size estimations, except *A. burtoni*, were taken from the animal genome size database [57], *A. burtoni *estimation from [50]. Size estimations of the D1 and D2 clusters were obtained from [46]. The gene flt4 is not included in this analysis. The graph includes the regression lines of the datasets if possible.

Our previous study [46] showed that the sequence of the cichlid D1 ParaHox paralogon again is of an intermediate length, being shorter than that of *D. rerio *(29%) but longer that the other D1 paralogons of the other species investigated (*O. latipes *102%, *T. nigroviridis *148%, *T. rubripes *140%, *G. aculeatus *125%). In both cases the mammalian sequence is the longest and the sequences of the pufferfishes the shortest (see Additional file 2). To test if this is a paralogon-specific effect or an effect of the different genome sizes of the various organisms, we plotted genome size versus cluster size. Figure 7 shows the comparisons (regression analyses) between the genome sizes of different organisms and the length of the C1, C2, D1 and D2 paralogons. The genome sizes of *Homo sapiens*, *Mus musculus, Danio rerio, Takifugu rubripes, Tetraodon nigroviridis, Gasterosteus aculeatus *and *Oryzias latipes *were taken from the animal genome size database [57]. The genome size estimate of *Astatotilapia burtoni *was obtained from reference [50].

While the C1 paralogons of teleosts show a similar genome size to cluster size relationship as the mammalian clusters, the C2 clusters are much more condensed but also show the same trend, namely that the cluster size is linked to overall genome size (Figure 7). The D1 paralogons are also much more condensed than the C1 paralogons, but they also display a linear relationship between genome and cluster size, including also the mammalian sequences. An obvious deviation from previously described pattern can be seen in the D2 paralogons of the pufferfishes. Relative to their very compact genome size, the D2 paralogon is surprisingly large. A possible reason for this could be that the maximal condensation of this cluster has already been reached and a further condensation might be detrimental in terms of selection. We can only speculate on this, but the minimum absolute size of the ParaHox paralogons might be determined by the necessary spatial relationships of the individual transcriptional units within these gene complexes and regulatory regions might need to be maintained at a minimal distance in the intergenic regions of adjacent genes in order to maintain the proper function of these genes. The *Astatotilapia burtoni *D2 ParaHox paralogon could not be included in this comparison because the gene *cdx1b *is not on the investigated BAC clone.

### Gene cluster breakup and gene retention after genome duplications

The FSGD provides the opportunity to study genomes following a whole genome duplication event [[33] and references therein]. For the Hox gene clusters of teleosts, it has been observed before [[39] and references therein] that, although all fish genomes studied so far vary in the gene content and even number of Hox gene clusters, the total number of Hox genes contained in their genomes is about the same as in the genomes of tetrapods, which did not experience this WGD. It has been suggested that particularly the Hox gene clusters are, typically, maintained more or less intact, because they are likely to be strongly regulated by sequential activation and cluster completeness is necessitated by corrected interdigitated gene control [58].

What seems remarkable as well is that the evolutionary forces keeping Hox gene number rather constant seem to be stronger than those that maintain the cohesion and physical linkage on chromosomes of individual clusters following a WGD. Mulley et al. [42] noted that the ParaHox cluster stayed intact in ancestral fish lineages such as *Amia *and *Polypterus*, yet noted the fragmentation of the ParaHox clusters in teleosts, that happened due to gene loss and not because of transpositions or inversions [42]. The FSGD duplicated all genomic regions including the clustered sets of homeobox genes such as Hox, ParaHox and NK. The selection pressures that maintained those clusters intact in part of the metazoans, seem to be relaxed, as for many of these gene clusters, several genes seem to have been lost [59], despite the fact that these, often apparently co-regulated arrays of genes, seem to share enhancers and are regulated in an interdigitated fashion (Figure 1). Mulley et al. [42] proposed that the maintenance of a gene cluster is based on interdigitated and/or shared enhancers. The FSGD duplicated not only the genes but also the enhancers and therefore might have released the need for a tight clustering. Our analysis of the ParaHox clusters in teleosts supports this idea in so far as the ParaHox clusters are broken up. Yet, the total number of six ParaHox genes is maintained in post duplication teleost genomes. If the comparison is extended to a larger paralogon than the set of ParaHox genes alone – as was done in this study -it becomes clear that in larger genomic regions there the constancy of gene numbers does not persist. Our analysis shows that some, although not all, additional duplicated genes flanking the ParaHox clusters were retained following a WGD (Figure 1). This might imply that different selective forces such as increased tolerance to more gene product, due to the doubled number of genes, or functional changes (sub-, neofunctionalization) of those genes might be acting. This finding might argue that although differential gene loss on different chromosomal regions is permitted following a WGD through genetic redundancy of *cis*-regulatory elements, the overall constancy of gene number is strongly selected for by balancing selection at least for transcription factors such as ParaHox genes. Balancing selection might be acting on *trans*-regulatory mechanisms to countact possibly negative effects of dosage differences. Moreover, possibly weaker selective forces against duplicate genes might permit the retention of probably not co-regulated genes outside of gene clusters after a WGD on one hand. It seems plausible that these different selective forces might also have to do with not only their arrangements in clusters, but also which kind of gene is duplicated (e.g., regulatory genes vs. housekeeping genes). Again, selection might act more strongly in bringing about the loss of interdigitated genes within cluster following a WGD to maintain a modal gene number per genome of these clustered homeobox genes in order to reduce potentially negative changes in dosage following a WGD. The fact, that the number of ParaHox genes before and after the FSGD remained unchanged, indicates a possibly strong regulatory gene dose restriction that would select for the rapid loss of "superfluous" genes. With the exception of the gene *cdx1 *no gene of the ancestral ParaHox cluster was retained in two copies. Possibly one of the two *cdx1 *genes may be compensating for the loss of *cdx2 *gene, hence the retention of two *cdx1 *genes (Figure 1b, [42]).

Recently Negre and Ruiz [60] have discovered a surprising diversity of Hox gene cluster architectures in different species of *Drosophila*. Since breaks and inversions were found not too infrequently, they argue that not the integrity and organization of Hox clusters is the strongest target of selection. Rather they argue that functional constraints on individual Hox genes might be acting more forcefully on genomes so that functional sets of homeobox genes are maintained in the genome, which are not necessarily physically linked with unbroken colinearity. Other studies showed that an intact cluster is only important for temporal and not for special colinearity. In *Drosophila *where development is so rapid that almost all the Hox genes are activated at the same time, the cluster is permitted to be interrupted [[61] and references therein]. Similar reasoning might explain the sitution we describe for "dissolved" ParaHox parologons. Their genomically fixed gene content and orientation in teleost genomes, but their dispersed distribution over seven instead of four chromosomal regions would support the hypothesis that overall gene content is more strongly selected for than the integrity of gene clusters.

## Conclusion

We demonstrated the orthologous relationship of the genes of the C and D ParaHox paralogons (Figure 2). Relative rate tests revealed that with the exception of one gene the a-copy always evolves more slowly than the b-copy, the exception being the ParaHox gene *cdx1*, where the b-copy evolves significantly slower. The relative rate tests also show that the C paralogons evolve more slowly than the D paralogons.

A mVista analysis of the D clusters was performed in an earlier study [46]. We found a number of conserved genomic regions in the C1 ParaHox paralogon that were located in intergenic regions. One conserved sequence block, located at the position 119–130 kb on the *A. burtoni *BAC clone 99M12, was confirmed to be another gene, the transmembrane protein *HPT-1*, by BLAST search. We also found evidence that the ParaHox paralogon of the pufferfishes is apparently close to the maximal possible reduction in size.

Despite having undergone an additional genome duplication the total number of ParaHox genes in the genome of teleost fish is maintained at six genes that are distributed over seven chromosomal regions instead of four as in the genomes of tetrapods. Other genes that are physically linked with the ParaHox genes in the same paralogon were also reduced in number following the FSGD. However, while typically ten of these are found in tetrapods 14 are maintained in teleost fish genomes. We discuss possible selective reasons for keeping modal numbers of homoebox genes constant throughout hundreds of millions of years of evolution while permitting to differentially loose ParaHox genes on some ParaHox paralogons.

Future research should include the description of possible binding sites in the conserved elements and functional studies of those putative regulatory elements found by *in silico *analyses.

## Methods

### BAC Library screening & Shotgun Sequencing

We previously constructed a BAC library of the East African haplochromine cichlid fish *Astatotilapia burtoni *[50]. This library was screened for *kita *positive clones with the *kita *specific primer set Burt-Kit-F-474/Burt-Kit_R-672 according to [46]. Using universal primers (gsh2_Ex1_For (AGAYCCCAGRAGATACCACT) and gsh2_Ex2.3_R (GTGCGCGCTCCTCTGGGTG)) designed on known teleost sequences, we confirmed the presence of the *gsh2 *gene on the BAC clone. The BAC plasmids of the recovered clones were extracted using the Large-Construct Kit (Qiagen) according to the manufacturer's manual and then sheared by sonification. The fraction of 2–3 kb was recovered from an agarose gel and blunt-end-ligated into the pUC18 vector of Roche and later electro-transformed into "Electro Max DH10B T1 Phage Resistant Cells" (Invitrogen). The subclones were grown in standard LB-medium (0.5 mg/ml ampicillin). The plasmid DNA was recovered using standard methods. The clones were sequenced directly using a standard M13F/M13R primer set on an ABI3100 automatic DNA sequencer (Applied Biosystems).

### Contig Assembly

The obtained sequences were quality trimmed by hand and checked for vector sequences using Sequencher 4.2 (Gene Codes Corporation). The same software was used for the contig assembly at the setting "dirty data", with a sequence similarity of 85% and an overlap of 20 bp. The full sequence of the genome of *E. coli *from the GENBANK database [52] was added to the analyses, so that all *E. coli *contaminated reads were filtered out of the assembly. Gaps between contigs were closed with gap spanning primers, designed with Primer3 [62]. For further analyses the remaining gaps were closed by 33 N's each.

The contigs of the BAC clone 99M12 were checked for corresponding forward/reverse clones in other contigs and a contig map was drawn. To check this map, the contigs were assembled into one single sequence according to the contig map. Using the tool bl2seq (align two sequences) (GENBANK database) [52], the contigs were BLAST-searched against chromosome 11 of *Tetraodon nigroviridis *containing its C1 ParaHox cluster. The contig map was then corrected using the information from the bl2seq analysis.

### Sequence Annotation

The ontology of the genes sequenced were determined by sequence comparisons with the available genomes of *Takifugu rubripe*s [63], (version 4.0), *Tetraodon nigroviridis *[64], (version 1–64), *Oryzias latipes *[65], (version 200506), *Gasterosteus aculeatus *[66], (version 41) and already annotated genes from *Danio rerio*, *Mus musculus *and *Homo sapiens *were taken from GENBANK [52]. The provided annotations of the *Homo sapiens*, *Mus musculus *and *Danio rerio *sequences from GENBANK database [52] were used to help to identify the intron/exon structure of the respective genes in *Takifugu rubripes*, *Tetraodon nigroviridis *and *Oryzias latipes*. In some cases the *Danio rerio *sequence could not be included into the analyses due to apparent miss-assemblies.

### Phylogenetic and Sequence Analyses

For phylogenetic analyses, Nexus files were processed via PAUP* [67] to eliminate positions that could not be aligned. The appropriate models of molecular evolution were estimated using the program modelgenerator [68]. Maximum likelihood trees and bootstrapping (1000 replicates) were calculated in PHYML [69]. Bayesian Inference was performed in Mr. Bayes 3.1 [70,71] (1,000,000 generations/5000 burnin).

Vista Plots were obtained via the mVista option on the Vista homepage [72]. The alignment program used was LAGAN (Global multiple alignment of finished sequences) [73]. For *Homo sapiens *the human/primate-specific RepeatMasker and for Mus musculus the mouse/rat/rodent specific RepeatMasker was used. For all other sequences the fugu-specific RepeatMasker was used as a stand in.

## Authors' contributions

This study was conceived by SH, WS, IB and AM. Most of the laboratory work was done by NS and IB. In silico analyses were conducted by NS with the help of SH, WS and IB. The manuscript was drafted by NS and read and revised by all authors.

## Supplementary Material

Additional file 1 **Location of the genes of the C1 and C2 ParaHox paralogon**. List of the genes used for the computation of the trees via PHYML and Mr. Bayes analyses, including the orientation of the gene (→ means 5'-3'; ← means 3'-5' orientation) and the database the DNA sequence was taken from. All sequences from NCBI (National Center for Biotechnology Information) [52] were taken as annotated there; the sequences from the other databases (MGP (Medaka Genome Project) [65], version 200506) and Ensembl [66]*T. nigroviridis *version Tetraodon7, *T. rubripes *version Fugu4.0, *O. latipes *version Medaka1, *G. aculeatus *version BroadS1,*D. rerio *version Zv6) were annotated by hand. Click here for file

Additional file 2 **Comparison of the genome size and the size of the C1, C2, D1 and D2 ParaHox paralogons**. All genome size estimations, except *A. burtoni *from the animal genome size database [57], *A. burtoni *estimation from (Lang et al. 2006); size estimation of the D1 and D2 cluster from (Braasch et al. 2006). The gene *flt4 *is not included in this analysis. Click here for file
