# Supplementary material for: Comparative genomics of ParaHox clusters of teleost fishes: gene cluster breakup and the retention of gene sets following whole genome duplications
Source: BMC Genomics. 2007 Sep 6;8:312. doi: 10.1186/1471-2164-8-312 (PMC2020491; doi:10.1186/1471-2164-8-312)
Supplement: Additional file 1 — Location of the genes of the C1 and C2 ParaHox paralogon. List of the genes used for the computation of the trees via PHYML and Mr. Bayes analyses, including the orientation of the gene (→ means 5'-3'; ← means 3'-5' orientation) and the database the DNA sequence was taken from. All sequences from NCBI (National Center for Biotechnology Information) [52] were taken as annotated there; the sequences from the other databases (MGP (Medaka Genome Project) [65], version 200506) and Ensembl [66]T. nigroviridis version Tetraodon7, T. rubripes version Fugu4.0, O. latipes version Medaka1, G. aculeatus version BroadS1,D. rerio version Zv6) were annotated by hand. [file 1471-2164-8-312-S1.doc]

## Table S1 - Location of the genes of the C1 and C2 ParaHox paralogon.

| **gene locus** | **organism** | **gene** | **location** | **orientation** | **annotation** | **database** |
| --- | --- | --- | --- | --- | --- | --- |
| **C(1)** | *Homo sapiens* | *gsh2* | chr4 | 🡪 | gi|50960714 | NCBI |
|  |  | *pdgfrα* | chr4 | 🡪 | gi\|61699224 | NCBI |
|  |  | *kit* | chr4 | 🡪 | gi\|4557694 | NCBI |
|  |  | *kdrb* | chr4 | 🡨 | gi\|11321596 | NCBI |
|  |  | *clock* | chr4 | 🡨 | gi\|25777594 | NCBI |
|  | *Mus musculus* | *gsh2* | chr5 | 🡪 | gi\|18921209 | NCBI |
|  |  | *pdgfrα* | chr5 | 🡪 | gi\|6755011 | NCBI |
|  |  | *kit* | chr5 | 🡪 | gi\|10947120 | NCBI |
|  |  | *kdrb* | chr5 | 🡨 | gi\|27777647 | NCBI |
|  |  | *clock* | chr5 | 🡨 | gi\|2114487? | NCBI |
|  | *Danio rerio* | *gsh2* | chr20 | 🡪 | gi\|92097946 | NCBI |
|  |  | *pdgfrα* | chr20 | 🡪 | gi\|34916051 | NCBI |
|  |  | *kita* | chr20 | 🡪 | gi\|18858926 | NCBI |
|  |  | *kdrb* | chr20 | 🡨 | gi\|109254770 | NCBI |
|  |  | *clock* | chr20 | 🡨 | gi\|18858444 | NCBI |
|  | *Tetraodon nigroviridis* | *gsh2* | chr1r | 🡪 | scaf15025 | Ensembl |
|  |  | *pdgfrα* | chr1r | 🡪 | scaf15025 | Ensembl |
|  |  | *kita* | chr1r | 🡪 | scaf15025 | Ensembl |
|  |  | *kdrb* | chr1r | 🡨 | scaf15025 | Ensembl |
|  |  | *clock* | chr1r | 🡨 | scaf15025 | Ensembl |
|  | *Takifugu rubripes* | *gsh2* | scaff13 | 🡪 | scaffold_13 | Ensembl |
|  |  | *pdgfrα* | scaff13 | 🡪 | scaffold_13 | Ensembl |
|  |  | *kita* | scaff13 | 🡪 | scaffold_13 | Ensembl |
|  |  | *kdrb* | scaff13 | 🡨 | scaffold_13 | Ensembl |
|  |  | *clock* | scaff13 | 🡨 | scaffold_13 | Ensembl |
|  | *Oryzias latipes* | *gsh2* | scaff1264 | 🡪 | scaffold1264 | MGP |
|  |  | *pdgfrα* | scaff1264 | 🡪 | scaffold1264 | MGP |
|  |  | *kita* | scaff578 | 🡪 | scaffold578_1 | MGP |
|  |  | *kdrb* | scaff578 | 🡨 | scaffold578 | MGP |
|  |  | *clock* | scaff2436 | 🡨 | scaffold2436 | MGP |
|  | *Gasterosteus acuelatus* | *gsh2* | groupVIII | 🡪 | scaff71, contig 10998 | Ensembl |
|  |  | *pdgfrα* | groupVIII | 🡪 | scaff71, contig 11000 | Ensembl |
|  |  | *kita* | groupVIII | 🡪 | scaff71, contig 11002 | Ensembl |
|  |  | *kdrb* | groupVIII | 🡨 | scaff71, contig 11007 | Ensembl |
| **C2** | *Danio rerio* | *kitb* | chr1 | 🡪 | gi|68354367 | NCBI |
|  |  | *clock3* | chr1 | 🡨 | gi\|30231241 | NCBI |
|  | *Tetraodon nigroviridis* | *kitb* | chr18 | 🡪 | scaf9219 | Ensembl |
|  |  | *clock3* | chr18 | 🡨 | scaf9219 | Ensembl |
|  | *Takifugu rubripes* | *kitb* | scaff563 | 🡪 | scaffold_563 | Ensembl |
|  |  | *clock3* | scaff563 | 🡨 | scaffold_563 | Ensembl |
|  | *Oryzias latipes* | *kitb* | chr1 | 🡪 | scaffold147 | Ensembl |
|  |  | *clock3* | chr1 | 🡨 | scaffold147 | Ensembl |
|  | *Gasterosteus acuelatus* | *kitb* | groupIX | 🡪 | scaff31, contig 8222 | Ensembl |
|  |  | *clock3* | groupIX | 🡨 | scaff31, contig 8223 | Ensembl |
| **D(1)** | *Homo sapiens* | *cdx1* | chr5 | 🡪 | gi|4502762 | NCBI |
|  |  | *pdgfrβ* | chr5 | 🡨 | gi\|68216043 | NCBI |
|  |  | *csf1r* | chr5 | 🡨 | gi\|27262658 | NCBI |
|  |  | *flt4* | chr5 | 🡨 | gi\|33667110 | NCBI |
|  | *Mus musculus* | *cdx1* | chr18 | 🡨 | gi\|46559385 | NCBI |
|  |  | *pdgfrβ* | chr18 | 🡪 | gi\|6679258 | NCBI |
|  |  | *csf1r* | chr18 | 🡪 | gi\|6681044 | NCBI |
|  |  | *flt4* | chr11 | 🡪 | gi\|6679812 | NCBI |
|  | *Danio rerio* | *cdx1* | chr14 | 🡪 | scaffold2007 | Ensembl |
|  |  | *pdgfrβ* | chr14 | 🡨 | scaffold2007 | Ensembl |
|  |  | *csf1r* | chr14 | 🡨 | scaffold2007 | Ensembl |
|  |  | *flt4* | chr14 | 🡨 | scaffold2002 | Ensembl |
|  | *Tetraodon nigroviridis* | *cdx1a* | chr1 | 🡨 | scaf14944 | Ensembl |
|  |  | *pdgfrβ1* | chr1 | 🡪 | scaf14944 | Ensembl |
|  |  | *csf1ra* | chr1 | 🡪 | scaf14944 | Ensembl |
|  |  | *flt4* | chr1 | 🡨 | scaf14573 | Ensembl |
|  | *Takifugu rubripes* | *cdx1a* | scaff203 | 🡨 | scaffold_203 | Ensembl |
|  |  | *pdgfrβ1* | scaff203 | 🡪 | scaffold_203 | Ensembl |
|  |  | *csf1ra* | scaff203 | 🡪 | scaffold_203 | Ensembl |
|  |  | *flt4* | scaff89 | 🡨 | scaffold_89 | Ensembl |
|  | *Oryzias latipes* | *cdx1a* | chr10 | 🡨 | scaffold147 | Ensembl |
|  |  | *pdgfrβ1* | chr10 | 🡪 | scaffold147 | Ensembl |
|  |  | *csf1ra* | chr10 | 🡪 | scaffold147 | Ensembl |
|  |  | *flt4* | chr10 | 🡪 | scaffold18 | Ensembl |
|  | *Gasterosteus acuelatus* | *cdx1* | groupIV | 🡨 | scaff17, contig 5889 | Ensembl |
|  |  | *pdgfrβ1* | groupIV | 🡪 | scaff17, contig 5889 | Ensembl |
|  |  | *csf1ra* | groupIV | 🡪 | scaff17, contig 5889 | Ensembl |
|  |  | *flt4* | groupIV | 🡪 | scaff17, contig 5932 | Ensembl |
| **D2** | *Tetraodon nigroviridis* | *cdx1b* | chr7 | 🡨 | scaf14536 | Ensembl |
|  |  | *pdgfrβ2* | chr7 | 🡪 | scaf14536 | Ensembl |
|  |  | *csf1rb* | chr7 | 🡪 | scaf14536 | Ensembl |
|  | *Takifugu rubripes* | *cdx1b* | scaff352 | 🡨 | scaffold_352 | Ensembl |
|  |  | *pdgfrβ2* | scaff51 | 🡪 | scaffold_51 | Ensembl |
|  |  | *csf1rb* | scaff51 | 🡪 | scaffold_51 | Ensembl |
|  | *Oryzias latipes* | *cdx1b* | chr14 | 🡨 | scaffold86 | Ensembl |
|  |  | *pdgfrβ2* | chr14 | 🡪 | scaffold86 | Ensembl |
|  |  | *csf1rb* | chr14 | 🡪 | scaffold86 | Ensembl |

List of the genes used for the computation of the trees via PHYML and Mr. Bayes analyses, including the orientation of the gene (🡪 means 5’-3’; 🡨 means 3’-5’ orientation) and the database the DNA sequence was taken from. All sequences from NCBI (National Center for Biotechnology Information) [52] were taken as annotated there; the sequences from the other databases (MGP (Medaka Genome Project) [63], version 200506) and Ensembl [64] *T. nigroviridis* version Tetraodon7, *T. rubripes* version Fugu4.0, *O. latipes* version Medaka1, *G. aculeatus* version BroadS1, *D. rerio* version Zv6) were annotated by hand.
