# Supplementary material for: Comparative genomics of ParaHox clusters of teleost fishes: gene cluster breakup and the retention of gene sets following whole genome duplications
Source: BMC Genomics. 2007 Sep 6;8:312. doi: 10.1186/1471-2164-8-312 (PMC2020491; doi:10.1186/1471-2164-8-312)
Supplement: Additional file 2 — Comparison of the genome size and the size of the C1, C2, D1 and D2 ParaHox paralogons. All genome size estimations, except A. burtoni from the animal genome size database [57], A. burtoni estimation from (Lang et al. 2006); size estimation of the D1 and D2 cluster from (Braasch et al. 2006). The gene flt4 is not included in this analysis. [file 1471-2164-8-312-S2.doc]

## Table S2 - Comparison of the genome size and the size of the C1, C2, D1 and D2 ParaHox paralogons.

| organism | genome size | | cluster size [kb] | | | | % of genome | | | |
| --- | --- | --- | --- | --- | --- | --- | --- | --- | --- | --- |
|  | [pg] | [Mb] | **C1** | C2 | D1 | D2 | **C1** | C2 | D1 | D2 |
| *H. sapiens* | 3.50 | 3,423 | **1379.36** | - | 129.6 | - | **0.0400** | - | 0.0038 | - |
| *M. musculus* | 3.30 | 3,227 | **1159.62** | - | 94.81 | - | **0.0360** | - | 0.0029 | - |
| *D. rerio* | 1.98 | 1,936 | **340.04** | 76.68 | 165.14 | - | **0.0180** | 0.0040 | 0.0085 | - |
| *T. rubripes* | 0.40 | 391 | **94.46** | 17.27 | 34.45 | 1034.54 | **0.0240** | 0.0044 | 0.0088 | 0.2646 |
| *T. nigroviridis* | 0.43 | 421 | **89.26** | 23.09 | 32.71 | 902.56 | **0.0210** | 0.0055 | 0.0078 | 0.2144 |
| *O. latipes* | 1.08 | 1,056 | **387.71** | 44.10 | 47.55 | 294.39 | **0.0370** | 0.0042 | 0.0045 | 0.0279 |
| *A. burtoni* | 0.97 | 949 | **133.56** | - | 48.33 | 83.37 | **0.0140** | - | 0.0051 | (0.0088) |
| *G. acuelatus* | 0.58 | 567 | **98.65** | 22.92 | 38.57 | - | **0.0170** | 0.0040 | 0.0068 | - |

All genome size estimations, except *A. burtoni* from the animal genome size database (www.genomesize.com), *A. burtoni* estimation from (Lang et al. 2006); size estimation of the D1 and D2 cluster from (Braasch et al. 2006). The gene *flt4* is not included in this analysis.
